# A modified supraorbital surgical approach for enucleation- exenteration in dromedary camels (camelus dromedarius): Clinical study

**DOI:** 10.1371/journal.pone.0306710

**Published:** 2024-08-29

**Authors:** Mohamed Marzok, Mohamed A. Nazih, Adel I. Almubarak, Thnaian A . Al-thnaian, Khalid M. Alkhodair, Mohamed Tharwat, Mohamed W. El-Sherif

**Affiliations:** 1 Department of Clinical Sciences, College of Veterinary Medicine, King Faisal University, Al-Ahsa, Saudi Arabia; 2 Department of Surgery, Faculty of Veterinary Medicine, Kafrelsheikh University, Kafrelsheikh, Egypt; 3 Department of Anatomy, Faculty of Veterinary Medicine, New Valley University, El-kharga, New Valley, Egypt; 4 Department of Anatomy, College of Veterinary Medicine, King Faisal University, Al-Ahsa, Saudi Arabia; 5 Department of Clinical Sciences, College of Veterinary Medicine, Qassim University, Buraidah, Saudi Arabia; 6 Department of Animal Medicine, Faculty of Veterinary Medicine, Zagazig University, Zagazig, Egypt; 7 Department of Surgery, Faculty of Veterinary Medicine, New Valley University, El-kharga, New Valley, Egypt; Rajasthan University of Veterinary and Animal Science, INDIA

## Abstract

Enucleation and exenteration are widely utilized ophthalmic procedures in veterinary field. Enucleation in camels is like other large animals, typically performed under the influence of heavy sedation and loco-regional analgesia. The aim of the current study was to introduce a new surgical approach to enucleate the eye of camels through supraorbital fossa approach. for that purpose, the technique was applied to seven camels referred to the King-fisal teaching veterinary hospital for unilateral enucleation. Assessment of applicability, safety and feasibility of this technique was done. All procedures were performed in the kush (sitting) position under the influence of heavy sedation with Xylazine HCl in combination with retrobulbar nerve block. A "C" shaped skin incision was made in the skin and fascia of the supraorbital fossa to enter the orbital cavity, after which the periorbital fat was gripped, dissected and removed. Bleeding controlled by electrocautery and visible large blood vessels were ligated. After ligation the optic nerve and ophthalmic blood vessels, the eyeball was dissected sharply and freed from the orbital bony attachment. Finally, the orbital fascia and skin were sutured with simple interrupted pattern separately. The approach proved successful in all camels, with the enucleation procedure being both feasible and easily performed. The mean surgical time was approximately 46.6±12.4 minutes. The minimal occurrence of short and long-term complications was encouraging, and the cosmetic outcomes were notably improved. The supraorbital approach is a safe and effective technique for camel ophthalmic surgery, showing advantages in exposure and minimal complications. Further research is needed for validation and broader clinical applications.

## Introduction

Enucleation and exenteration are widely used ophthalmic procedures in veterinary practice that involves the complete surgical removal of the eye, or of all contents within the orbital cavity respectively [1, 2]. The indications are to relieve chronic pain, remove tumors and control neoplasia metastases, and address severe trauma that are not managed surgically and to address severe inflammation/ infection that cannot treated medically [3–8]. The two surgical enucleation techniques are the transconjunctival [7, 9] and transpalpebral approaches [5, 7, 10, 11]. The main goals of the procedure are to remove the ocular structures with minimal intra and postoperative complications [7, 8], and to maintain cosmetic appearance [7, 12, 13] through implantation of orbital prosthesis to maintain the shape and size of the orbital socket [13]. Several congenital [14] and acquired ocular disorders have been reported in camels with an incidence rate up to 12% of the studied sample [15]. Primary or secondary keratoconjunctivitis and nonulcerative keratitis, always managed medically without the need of surgical intervention [15–17]. However, perforating wounds of the cornea and intra or extraoral tumors of the ocular structures have an incidence rate up to 25% of investigated ocular conditions and need surgical enucleation [15–19]. Enucleation is mostly safe procedure, but potential postoperative complications include bleeding, infection, wound dehiscence, meningitis, and blindness of the contralateral eye [3, 4, 20]. Like other animals, enucleation in camels performed mostly by the transpalpebral technique [21, 22]. The unique anatomy of the camels’ head, characterized by an extensive large temporal fossa that extends rostrally to large depression, the orbital fossa. The latter formed an oval bony depression lined with the zygomatic and orbital processes and acts as a dorsal window to the orbital cavity [23, 24]. In recent years, there has been a growing interest in developing new surgical techniques for the treatment of eye diseases in camels. Thus, the objective of the current study is to introduce a novel surgical enucleation approach through the orbital fossa that allows for the optimal navigation of the orbital contents and operating of the orbital tissues and assess its feasibility and safety.

## Materials and methods

The current study was a clinical study reviewed, permitted, and approved by the Research Ethics Committee (REC) of King Faisal University, KSA (Approval NO. KFU-REC/2024- ETHICS 2,090). The present study was conducted on live camels presented for surgical intervention. All participating camels underwent complete recovery and were subsequently discharged. Prior to surgery, informed consent was obtained from the owners for both the surgical procedure and the anonymized publication of data. All methods were carried out in accordance with relevant guidelines and regulations. All methods are reported in accordance with ARRIVE guidelines.

### Ultrasound imaging

Ultrasound imaging of the eye and orbital cavity was performed to evaluate the extent of the procedure. The imaging was performed using an ultrasound machine equipped with a high-frequency micro convex transducer 7.5–9 MHz (*Mindray DP10-Mindray*, *China*). Two acoustic windows were used. In the first one, the transducer was positioned trans corneal on a coupling gel pad on the eyelids, and subsequent images were analyzed to ascertain the location and dimensions of the eyeball in comparison with the orbital cavity. Other images were obtained through the second window by placing the transducer in the supra orbital fossa. The integument covering the temporoparietal fossa extends over the superficial temporal fascia externally. This robust layer envelops the supra-orbital fossa, extending both rostrally and dorsally in conjunction with the frontal fascia. Ventral attachment occurs at the temporal crest, providing anchorage for the frontal part of the external auricular muscles and the Malaris muscle. In deeper layers, the thinner deep temporoparietal fascia envelops the temporalis fossa and its contents. On the external surface of this fascia, the palpebral branch of the auriculo-palpebral nerve of the facial nerve courses rostrally, passing over the Malaris muscle. The hair in the orbital fossa was clipped, and a coupling gel was then applied. A micro convex array transducer (endocavity probe) was used. Long and short axis images from the posterior of the eye were analyzed.

### Surgical procedure

The current study was reviewed, permitted, and approved by the Research Ethics Committee (REC) of King Faisal University, KSA (Approval NO. KFU-REC/2024- ETHICS 2,090). All methods were carried out in accordance with relevant guidelines and regulations. All methods are reported in accordance with ARRIVE guidelines. Seven adult male camels (median age: 7 ± 1.5 years, median weight: 510 ± 13.7 kg) were admitted to the surgery clinic of the teaching hospital at the College of Veterinary Medicine, King Faisal University, KSA. These camels exhibited unilateral perforating corneal wounds (two camels in the right eye and one camel in the left eye), while four camels presented with deep ulcerative keratitis (two in the left eye and two in the right eye) with varying degrees of severity, including corneal opacity, complete blindness, and pus formation. The injuries were attributed to physical trauma during transportation, and previous attempts at medical treatment had been unsuccessful. Local veterinarians referred these cases to the teaching hospital for surgical enucleation. Prior surgery, camels were deprived of food but not water for 12 hours. A single intramuscular injection of Amoxicillin trihydrate 7.5 mg/kg (*AmoxyKel 15 L*.*A*. *Kela*, *Belgium*) was administered one hour before surgery, and intravenous shot of flunixin meglumine 2mg/kg (*Finadyne*, *MSD*, *USA*) intravenous administered just before sedation.

Surgical procedures were performed in the kush (sitting) position. After physical restraint with ropes, a 14-gauge intravenous catheter was placed in the jugular vein. Camels were sedated by intravenous administration of Xylazine HCl at a dose of 0.4 mg/kg (*Xylazinbio 2%*, *Bioveta*, *Ankara*, *Turkey*). The neck was extended and positioned on a soft, padded surgical stand. Subsequently, the region from the temporal fossae to the upper eyelid was clipped, aseptically prepared, and covered with a double-layered four-corner drape, ensuring a window over the orbital fossa. Anesthesia of the ocular structures was achieved via retrobulbar block. A18-gauge, 150 mm spinal needle was inserted in the skin just anterior to the point where the supraorbital process joins the zygomatic arch at angle of about 30 degrees with the horizontal zygomatic arch. The needle is directed caudally and ventrally to pass in front of the anterior border of the coronoid process of the mandible and pushed deeply until it hits the floor of the pterygopalatine fossa and 10 ml of 2% lidocaine (*Lidocaine*, *Hospira Inc*., *USA*) solution is deposited at a depth of 8–12 cm [25].

**Fig 1:**
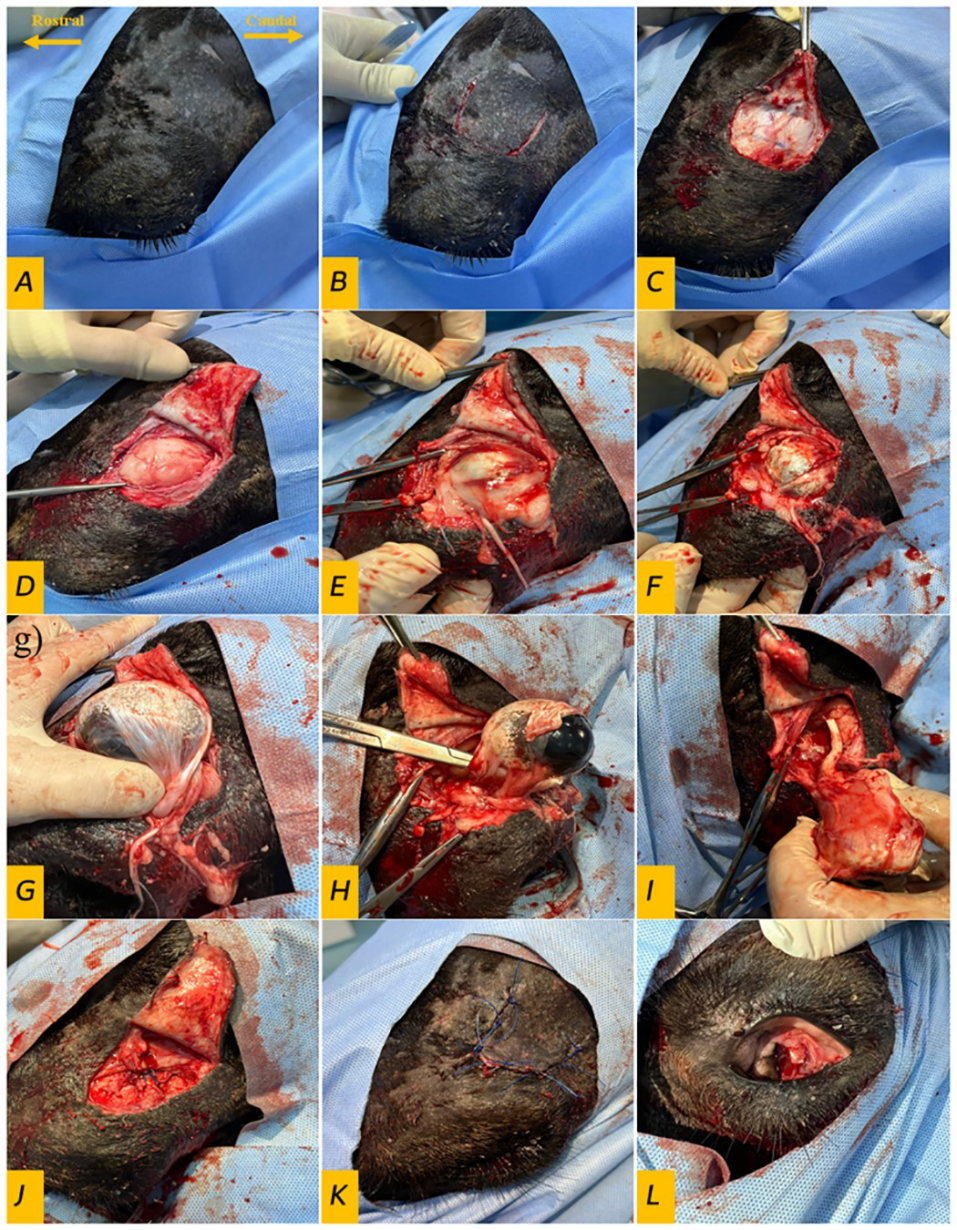
The orbital fossa enucleation approach. **A)** aseptic preparation of the orbital fossa, **B)** skin incision, **C)** dissection and creation of skin flap, **D)** dissection of the supraorbital facia and exposure of the periorbital fat, **E)** dissection and excision of the periorbital fat, **F)** dissection and posterior attachment of the eyeball with the bony orbit, **G)** dissection of the anterior bulbar attachment of the eyeball and bulbar conjunctive, **H)** dissection of extraocular muscular attachment, **I)** removal of the eye, **J)** suture of the supraorbital fascia, **K)** closure of the skin incision, and **L)** after surgery, the eyelids preserved intact.

With scalpel, a "C" shaped skin incision is made, followed by dissection to create a skin flap. Similar incision is made in the orbital fascia. The flap created with its free end posterior flipped backward. Once the orbital window is created, the periorbital fat is exposed. With grasping forceps, the orbital fat gripped and dissected with curved mayo scissors. Bleeding controlled by touching the oozing spots with monopolar electrode. Visible blood vessels were ligated with number 3–0 polyglactin suture (*Vicryl*, *Ethicon*, *USA*). After removal of the orbital fat, the orbital cavity extensively irrigated with sterile saline solution repeatedly until the orbital content became clear. A 20 cm mayo needle holder is used to pass a needle with number 3–0 polyglactin suture (*Vicryl*, *Ethicon*, *USA*) around the most posterior part of the tenons capsule to ligate the optic nerve and ophthalmic blood vessels. Another ligature was placed 0.5 cm apart, and the capsule then severed with 20 cm curved Metzenbaum scissor from in-between. After freeing the posterior attachment of the tenons capsule, the freed tail was grasped with forceps while dissecting the ocular tissue from the orbital bony attachment. After freeing the eyeball, the non-dominant hand of the surgeon used to push the eyeball caudally into the orbital cavity concurrently with sharp dissection of the bulbar conjunctive with curved Metzenbaum scissors. Bleeding was controlled by the application of mosquito hemostats, followed by large vessels ligation. The orbital socket packed with sterile gauze and the skin flap replaced to normal place. The orbital fascia was then sutured with simple interrupted pattern using number 2–0 polyglactin sutures (*Vicryl*, *Ethicon*, *USA*) and the skin was closed using a simple interrupted pattern with number 0 polypropylene sutures (*Prolene*, *Ethicon*, *USA*). The day after day dressing regimen involved replacing the used gauze with sterile gauze partially soaked in povidone-iodine solution, followed by application of 10% povidone-iodine solution to the orbital socket and spraying with an antibiotic. The surgical procedure steps are illustrated in Fig 1. The surgical procedures were consistently performed by the same surgeon, adhering to standardized procedural steps.

### Postoperative care and follow up

The animals were subjected to a short-term postoperative observation period of 24 hours to mitigate any potential complications arising from the surgical procedure. Then, the camels underwent a period of home-based veterinary care lasting 1 month to assess long-term outcomes and identify potential complications. Standardized wound care was performed, involving disinfection of the wound using povidone iodine solution, followed by the application of oxytetracycline-gentian violet spray and adhesive dressing. In addition, during the first three days, the camels received intramuscular Amoxicillin trihydrate 7.5 mg/kg twice daily (*AmoxyKel 15 L*.*A*. *Kela*, *Belgium*) and flunixin meglumine (*Finadyne*, *MSD*, *USA*) 2 mg/ kg intravenous injection.

### Evaluating the modified enucleation approach

The evaluation criteria included feasibility, safety, efficacy, surgical time, success rate, complication rate, operator experience, functional outcomes, cosmetic appearance, and long-term outcomes. Feasibility pertains to the practical viability of executing the technique, whereas safety evaluates the capacity to mitigate risks for both the patient and the operator. The intensity of hemorrhage, as described on a visual estimation [26, 27], whether mild, moderate, or severe, was determined by evaluating the volume and rate of blood loss, along with the efforts required for its control. Mild hemorrhage typically signifies the oozing of a small volume of capillary blood, easily managed by touching with a monopolar electrode. Moderate hemorrhage indicates a larger volume of blood loss (5–10 ml) originating from vascular sources, necessitating additional time and effort for management, including irrigation, identification of the vessel, and ligation. Severe hemorrhage denotes the loss of a substantial volume of blood from a single site (more than 10 ml), requiring immediate intervention such as packing to staunch the bleeding, followed by irrigation and ligation or cautery to achieve hemostasis. The volume of blood loss was also visually estimated based on the amount collected in the suction jar and the saturation and spread of blood on the surgical drape. Efficacy gauges the procedure’s success in attaining the intended outcomes. Other factors encompass the duration of the surgical procedure, calculated from the initial skin incision to the final suture applied. Success and complication rates, the influence of operator experience, functional outcomes, cosmetic appearance, and a comparative evaluation against established techniques are also crucial considerations. Additionally, the assessment extends to long-term outcomes, ensuring a comprehensive understanding of the sustained effectiveness and safety of the technique over an extended duration.

Statistical analysis was conducted using the standard error of means (SEM) and performed with SPSS software version 29.0.10 (IBM, USA).

## Results

### Ultrasonographic findings

#### Trans corneal ultrasonographic findings

**Fig 2:**
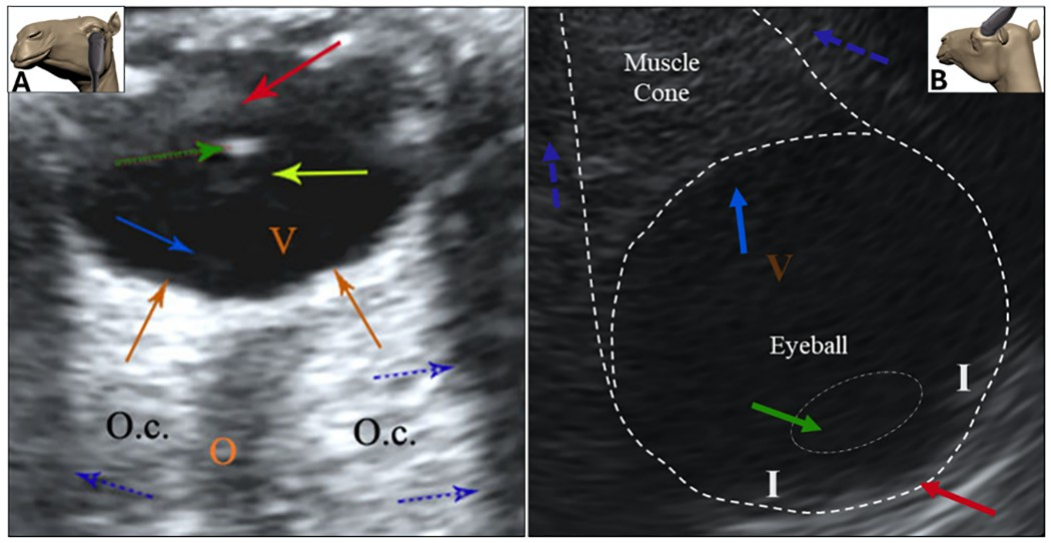
Photographs showing the trans corneal (A) and retrobulbar (B) ultrasonographic findings. C) cornea, lc) lens capsule, l) lens, i) iris, ac) anterior chamber, od) optic disc, oc) ocular muscle cone 1) vitreous body, 2) optic nerve, 3) optic foramen boundary, 4) orbital fat.

The ultrasonographic examination of the orbital contents explains the anatomical structures of the eyeball, periorbital cone, and the optic nerve. In the eyeball, it shows the anterior chamber, it appears as fusiform area occupies the rostral aspect of the eye lens and iris. The eye lens showed caudally to the anterior chamber as hyperechoic ovoid structure. It is surrounded by a very thin hypoechoic layer, the lens capsule. The intraocular cavity is filled by the vitreous body, which appeared as anechoic ovoid mass encloses the eye lens rostrally, the retina laterally and caudally. It appears homogeneous except at the circumscribed elliptical spot, the optic papilla. The optic nerve passes as hypoechoic cord extends within the orbital cone. Peribulbar ultrasonography is illustrated in Fig 2A.

#### Retrobulbar ultrasonographic findings

The ultrasonographic examination of the eye from the retrobulbar acoustic window showed fewer ocular details than the peribulbar view. The optic nerve and muscular cone of the eye viewed as homogeneous hyperechoic with less details. The eyeball showed a more circular hypoechoic structure. Retina is not well defined. Iris is evident in this view as hyper echoic structure. The eye lens is homogeneous, not well defined from the surrounding and the capsule not clear. The ultrasonography findings from the palpebral window showed the entire globe. Retrobulbar ultrasonography is shown in Fig 2B.

### Surgical procedure findings

Seven adult male dromedary camels, averaging (7±1.5) years in age and with a mean body weight of (510±13.7) kg, underwent orbital enucleation. These camels were referred to the hospital due to unilateral chronic septic ulcerative keratoconjunctivitis, and perforated cornea and damage of the eye lens due to physical trauma; four had the condition in the right eye, and three in the left eye. All camels had a history of unsuccessful medical treatment.

The procedure was applicable, with intraoperative complications limited to mild to moderate bleeding, all of which were effectively controlled using monopolar electrocautery and suture ligation. Hemorrhage occurred at specific anatomical sites during the surgical procedure. Bleeding from the skin and fascia incisions was minimal and managed through electrocautery. Moderate bleeding arose during excision of the orbital fat body, necessitating identification of the bleeding vessel and subsequent ligation. Similarly, dissection of the eyeball with the muscle cone resulted in moderate bleeding, which was effectively controlled through a combination of ligation and electrocautery techniques. No other intraoperative complications or adverse events were observed. The efficacy of the technique was demonstrated by its successful achievement of the surgical goal in all cases. Additionally, the procedure provided the surgeon with better exposure to the surgical field, allowing for more efficient manipulation and tissue handling. The mean surgical time was 49.6±3.25 minutes. Minimal edema and bleeding were observed in the first 38±5.6 hours postoperative. Afterwards, no postoperative bleeding or infection occurred, as evidenced by the absence of clinical signs such as redness, swelling, heat, pain, exudation, and fever. The decision to keep the eyelids open contributed to better wound drainage. Long-term outcomes demonstrated positive results, with no complications recorded, and healthy granulation tissue filling the ocular wound without any evidence of infection evidenced by clinical signs or general health issues.

## Discussion

The present study introduces a novel supraorbital enucleation approach in camels, aiming to optimize navigation within the orbital cavity and facilitate operating within the orbital cavity. The findings of this study demonstrated positive outcomes across various evaluation categories, which support the feasibility, safety, and efficacy of the technique. This new approach has been successfully performed primarily in an initial pilot study conducted on a cadaveric subject.

Enucleation commonly performed for incurable ophthalmic conditions [1, 2, 10, 15, 28]. Consistent with prior findings [21], performing the current technique on a physically restrained camel under deep sedation and retrobulbar anesthesia proved to be sufficient, offering ample surgical time to successfully complete the interventions. In contrast to previous reports [11, 12], no need to specific instruments and the general surgery tools were enough to finalize the surgery successfully. This ease of application contributes to the practicality and accessibility of the procedure in clinical settings [29, 30]. Safety considerations are paramount in any surgical technique [29]. The findings of the present study unveiled several safety indicators. Firstly, no vital structures were identified along the incision line. Secondly, the feasibility of controlling bleeding was demonstrated. Thirdly, keeping the eyelids open was shown to enhance wound drainage, and it minimized the traction force applied to the optic nerve.

The choice of the supraorbital fossa as a surgical window for enucleation in camels was based on its extensive entry boundaries [31], which provide appropriate access to the orbital structures. Detailed biometric analysis, derived from surgical anatomy and computed tomographic scans, supports this choice. Specifically, the mean diameter of the orbital fossa was found to be 4.04 cm, while the mean diameter of the orbit rim was 5.24 cm. Additionally, the mean diameter of the eyeball measured 3.32 cm, and the mean diameter of the deep orbital cavity was 4.62 cm. The mean retrobulbar area diameter was 2.98 cm. These dimensions collectively indicate that the supraorbital fossa offers sufficient space for the effective and safe surgical removal of the eyeball. Thus, the supraorbital approach is advantageous due to its anatomical compatibility with the required surgical procedures.

The efficacy of the technique was evident through the successful achievement of the surgical goal in all cases. The improved exposure provided by the novel approach facilitated better manipulation and tissue handling, enhancing the surgeon’s ability to perform the procedure effectively. The mean surgical time of approximately 49.6±3.25 minutes suggests that the novel technique is reasonably efficient and comparable to previous reports in small and large animals [7, 30]. The short-term complications observed during intra, and early postoperative phases align with those documented in previous reports, primarily characterized by minimal to intermediate bleeding [1, 7, 21]. There was no long-term complication recorded unlike reported in other techniques [1]. This is attributed to several factors. Firstly, the effective removal of a significant amount of orbital soft tissue minimizes postoperative hemorrhage, hematoma, and cyst formation. Secondly, maintaining the integrity and openness of the eyelids facilitates optimal drainage at the surgical site. Lastly, the optimal ability to obliterate dead space post-surgery is achieved using sterile packs inserted through the supraorbital surgical incision. Preserving the eyelids open not only contributed to better wound drainage, but also contributed to better cosmetic outcomes as recommended in the subconjunctival approach [7]. An ongoing study by the authors of this manuscript is evaluating the cosmetic outcomes following the implantation of orbital prosthesis post-enucleation using the orbital fossa approach. We hypothesize that this approach provides the surgeon with enhanced precision in fitting the prosthetic through the supraorbital incision. Additionally, the surgical approach empowers the surgeon to anchor the prosthesis securely to the bony orbit, thereby minimizing the risk of future displacement. Finally, the modified supraorbital enucleation-exenteration technique appeared to require minimal operator experience. The improved exposure of the surgical field facilitated easier access, which may reduce the learning curve for surgeons adopting the new approach.

## Conclusions

The supraorbital enucleation technique proves to be a safe, efficient, and effective approach for ophthalmic surgery in camels. Its advantages in exposure, tissue handling, and minimal complications contribute to its value in the existing literature. Nevertheless, additional research and larger-scale studies are advisable to validate these findings, explore broader clinical applications and to assess the long-term effects of this new technique on the welfare and productivity of camels.
